# Microbial Polyhydroxyalkanoates Granules: An Approach Targeting Biopolymer for Medical Applications and Developing Bone Scaffolds

**DOI:** 10.3390/molecules26040860

**Published:** 2021-02-06

**Authors:** Moushmi Goswami, Pavni Rekhi, Mousumi Debnath, Seeram Ramakrishna

**Affiliations:** 1 Department of Biosciences, Manipal University Jaipur, Rajasthan 303007, India; moushmi.181002001@muj.manipal.edu (M.G.); pavni.181002018@muj.manipal.edu (P.R.); 2 Department of Mechanical Engineering, National University of Singapore, Singapore 119260, Singapore; seeram@nus.edu.sg

**Keywords:** polyhydroxyalkanoates, PHA, biopolymer, biodegradable, bone scaffold, nanocomposite

## Abstract

Microbial polyhydroxyalkanoates (PHA) are proteinaceous storage granules ranging from 100 nm to 500 nm. *Bacillus* sp. serve as unique bioplastic sources of short-chain length and medium-chain length PHA showcasing properties such as biodegradability, thermostability, and appreciable mechanical strength. The PHA can be enhanced by adding functional groups to make it a more industrially useful biomaterial. PHA blends with hydroxyapatite to form nanocomposites with desirable features of compressibility. The reinforced matrices result in nanocomposites that possess significantly improved mechanical and thermal properties both in solid and melt states along with enhanced gas barrier properties compared to conventional filler composites. These superior qualities extend the polymeric composites’ applications to aggressive environments where the neat polymers are likely to fail. This nanocomposite can be used in different industries as nanofillers, drug carriers for packaging essential hormones and microcapsules, etc. For fabricating a bone scaffold, electrospun nanofibrils made from biocomposite of hydroxyapatite and polyhydroxy butyrate, a form of PHA, can be incorporated with the targeted tissue. The other methods for making a polymer scaffold, includes gas foaming, lyophilization, sol–gel, and solvent casting method. In this review, PHA as a sustainable eco-friendly NextGen biomaterial from bacterial sources especially *Bacillus cereus,* and its application for fabricating bone scaffold using different strategies for bone regeneration have been discussed.

## 1. Introduction

Polyhydroxyalkanoates (PHA) are microbial polymers present intracellularly and highly regarded as a NextGen biopolymer. Due to its significant attributes such as biodegradability, biostability, and compatibility along with exquisite physiochemical properties, it is considered as a replacement for synthetic plastics [1]. PHA offers mankind a unique opportunity for dealing with environmental issues such as non-recyclable waste burden, cheap and reliable alternative to conventional polymers [2]. Biodegradability, apart from thermal, mechanical, and chemical stability, is one of its prime features for its immense use in disciplines such as medicine, electronics, agriculture, food, and drug delivery [3].

PHA can be isolated from microorganisms belonging to multiple domains such as eubacteria, archaebacteria, and bacteria. Biogenesis of PHA is aided by a close-knit network of enzymes such as acetyl-CoA-acetyltransferases, acetoacetyl-CoA-reductases, PHA synthases [4], and a gene cluster involving phaC, phaM, phaZ, etc. The research on this biopolymer has focused on the isolation of PHA proteinaceous granules accumulating in microbial bodies and optimizing culture conditions contributing to increment in yield and biomass production, which helps to identify the potential source of PHA [5,6]. The success of the PHA as a sustainable nanocomposite in various medical applications and product commercialization needs a clear understanding of its microbial source of production, industrialization, downstream processing, and utilization of PHA and its nanocomposites [1]. In this review, we referred to published literature and tried to emphasize the journey of the polyhydroxyalkanoates, commonly known as PHA, from its microbial source with special reference to *Bacillus* species to its application as a PHA nanocomposite in different domains of medical applications, including its contribution as an important component in bone regeneration and bone scaffold.

## 2. PHA: A Biodegradable Microbial Biopolymer Synthesized as a Component of a Microbial Cell

PHA is synthesized by microorganisms as reserve material to provide the cell energy and carbon under unfavorable conditions and growth-limiting factors. These PHA granules are well-organized complex molecules, also designated as carbonosomes. PHA granules are layered by differently functioning polypeptides, therefore, appearing as inclusion bodies or granules. Among other proteins found on the outer layer of a PHA granule, a group of proteins known as “phasins” are also present covering almost 3/4th of the granular space. It has a vital function to play for maintaining the spherical stature of the PHA granule and also participates in cellular metabolism. Usually, the size of a PHA granule is highly variable and ranges from 100–500 nm depending on the type of PHA producing organism and phasins [7,8]. Its versatility helps in the synthesis and depolymerizing of PHA molecules. Phasins are of high importance especially in biomedical applications because of their inherent ability of strong binding affinity to protein tags and also to PHA molecules, making them easily engineerable and helping in producing and performing biotechnological products for in vitro experiments [9]. Phasin protein is an important factor required to produce the nano sized PHA. The granular nature of the nano sized PHA can be visualized using atomic force microscopy and the size difference can also be easily detected [10]. A phase-contrast light microscope can be useful to check the microscopic features inside the living bacterial cell as a bright structure, whereas a fluorescent microscope can make a distinction using fluorochromes, but the transmission electron microscope can distinguish the differences between the sizes of the granules inside the bacterial cell [11]. The characterization of the PHA granule can be performed using Fourier transform infrared spectroscopy, differential scanning calorimetry, thermal gravimetric analysis, and X-ray diffraction, respectively. Size-exclusion chromatography is advised to understand the polydispersity index, the number-average molar mass, and weight-average molar mass (Mw) [5,11,12]. The PHA polymer can be further studied using proton nuclear magnetic resonance spectroscopy and ^13^C-NMR spectra [5,12]. 

PHA granules are also governed by a cluster of genes working together for its biogenesis, functioning, and degradation. PhaC is highly important because it translates for PHA synthase enzyme, a major enzyme that is required to form R-hydroxyalkanoic acid from intermediates as a final product during the synthetic pathway. For the proper functioning and maximum activity of the PHA synthases another gene, PhaM is involved, first reported in *Ralstonia eutropha* [13]. PhaJ gene translates R-specific enoyl-CoA hydratase (R-hydratase), which again contributes to the PHA metabolism [14]. PhaZ gene works opposite to PhaC gene by translating PHA depolymerases involved in the degradation of short-chain length PHA [15]. 

### Bacillus as Novel PHA Producer

After the discovery of the first identified PHA molecules in *R. eutropha* [16], several microbial sources have been investigated for the accumulation of PHA. Out of all the microbial species isolated and searched, maximum research has been performed on *Bacillus* genera. Being versatile in nature, *Bacillus* sp. are found almost in every habitat ranging from extreme climates to temperate regions of the world. Moreover, because *Bacillus* is extensively studied for its structural and functional aspects, it is easier to clone their genes [17]. For this review, we focused on *Bacillus* species with special mention to the multiple strains of *Bacillus cereus*. Eight organisms were taken into consideration from which there were six strains of *B. cereus*, and they were compared for their content of PHA (Table 1). A comparison between the PHA yields before and after optimization methods revealed the high importance of using optimization of the nutrients and culture conditions for the PHA production. After comparing the yield of PHA among the *B. cereus* strains, the next step of the analysis was undertaken to find the genetic diversity and relatedness among the *B. cereus* strains and identify the most reliable *Bacillus* species/strains that can be determinative for increased PHA efficacy and most suitable for scale-up processes. 

**Figure 1:**
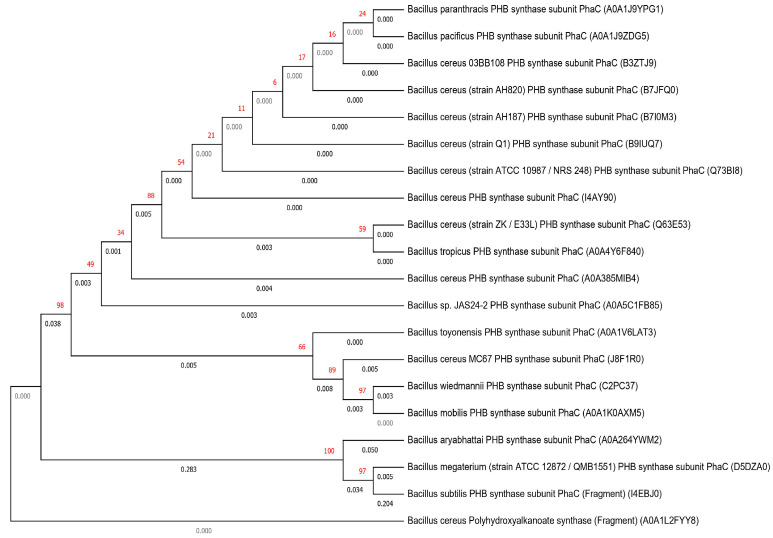
Taxonomic interrelationship among the different *Bacillus* species—phylogenetic tree constructed using the neighbor-joining method based on PhaC gene found in different *Bacillus* species sequenced on Uniprot database.

To analyze the relationship and relatedness of other *Bacillus* species with each other further, a phylogenetic tree was constructed (Figure 1) using nucleotide sequences of the PhaC gene found in the different *Bacillus* genera. The tree was constructed using MEGA X software [24] and the neighbor-joining method [25]. The tree was bootstrapped at 500 replicates [26], and each branch’s value and branch lengths are mentioned in Figure 1. Considering the polyhydroxyalkanoate synthase (Fragment) as the root branch and branch’s lengths, the phylogenetic tree indicates that there exist minor differences among the mentioned species. However, among them, there were three species, namely, *B. aryabhattai, B. megaterium,* and *B. subtilis* that showed maximum evolutionary distances from the root branch. Therefore, it can be concluded that the microbial origin with novel properties of the PHA granule production, was present univocally in all the mentioned *Bacillus* species and they all showcased the efficacy for PHA biopolymer production. However, it also gave a ray of hope that with culture optimization or using genetic recombination of PhaC gene from these closely associated *B. cereus* species, high yielding new variants could be used for increasing the yield of PHA.

## 3. Structural Diversity of PHA 

**Figure 2:**
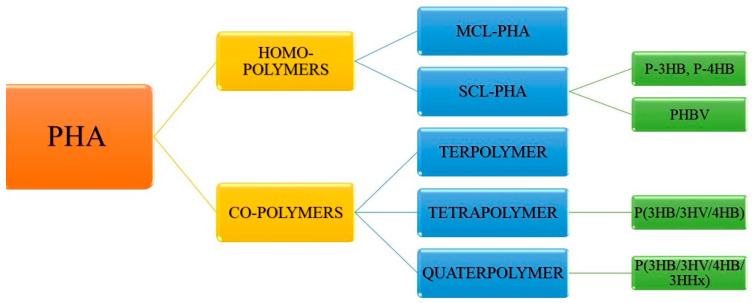
Classification helps in categorizing PHA into homopolymers and co-polymers. PHA: polyhydroxyalkanoate; PHB/P3HB: poly (3- hydroxybutyrate); PHBV: poly (3-hydroxybutyric acid-co-3-hydroxyvaleric acid)/polyhydroxybutyrate-co-valerate; P4HB: poly-4-hydroxybutyrate; MCL-PHA: medium-chain length polyhydroxyalkanoate; SCL-PHA: short-chain length polyhydroxyalkanoates.

Classified on the basis of the number of carbon atoms and their chain length, PHAs are usually medium-chain length PHAs (MCL-PHAs) and short-chain length PHAs (SCL-PHAs) (Figure 2). With the variation in the chain length, it is often observed that there is variability in crystallinity, elongation capacity, and tensile strength in this polymer. MCL-PHAs are crystalline polymers, elastomeric in nature and thus are different in physical properties from small-chain length PHAs. They are more flexible in nature, less crystalline (25%) with low melting temperature (ranging between 42–65 °C). On the other hand, SCL-PHAs are stiff, brittle, and highly crystalline in nature [27]. MCL-PHAs are known as true elastomers due to lower melting points. Once the temperature rises above Tm, they become sticky and amorphous [28]. β oxidation pathway is utilized by bacteria to produce MCL-PHA. The main substrate is acyl CoA, which is released after each cycle by utilizing two carbon atoms; 2-trans-enoyl-coa is converted to R-3-hydroxyacyl-coa by PhaJ (hydrolase enzyme) and then PhaC polymerizes R-3-hydroacyl-coa to MCL-PHA. Thiolases and dehydrogenase enzymes are important in catalyzing the two steps at the end of β oxidation pathway. These two steps have been researched extensively for accumulation and functional PHA increment [29]. Several fermenting strategies are applied by using co-substates such as decanoic acid (DA), derived sugars as studied recently by Oliveira et al. [30]. *Pseudomonas* sp. are the main producers of MCL-PHA [31]. They are capable of metabolizing all types of sugars except glucose. Therefore, hexoses are provided in access when both the sugars are provided as mixture. This results in faster metabolism of glucose [32]. Structurally, MCL-PHA is dependent on the type of carbon source provided. In *Pseudomonas* sp., MCL-PHA is produced by two different pathways—β oxidation when substrates such as alkanoic acid are provided and de novo fatty acid biosynthesis pathway when structurally unrelated substrates such as carbohydrates are provided [30].

SCL-PHA is one of the most industrially important classes of PHAs. SCL-PHA containing up to five carbon atoms in its monomer or repetitive units and is a good substitute biomaterial to conventional plastics. One of the well-researched SCL-PHAs is poly (3- hydroxybutyrate) [P3HB/PHB]. It is thermoplastic in nature and used in packaging and medical utilities [33]. Yet it is brittle and has high melting temperatures, therefore, limiting it to industrial applications [34]. Another SCL-PHA, poly-4-hydroxybutyrate (P4HB) has come out as a better alternative than P3HB and Poly(3-hydroxybutyric acid-co-3-hydroxyvaleric acid)/polyhydroxy butyrate-co-valerate (PHBV) due to its structurally strong nature and its elongation capability up to 1000%. This proves its higher flexibility and stretchability [35]. 

The polymerization of the monomeric components of the PHA nano-sized granule depends on the activity of PHA synthase [36] This helps to achieve the desired high molecular weight of the resultant PHA homopolymer and copolymers. The gel permeation chromatography results showed that in marine bacteria, the number average molecular weight of the PHAs after purification ranged from 3 × 10^3^–9.94 × 10^5^ g/mol [37]. The molecular weight of the PHA of 1 × 10^6^ g/mol was reported from certain higher plants and *Escherichia coli* and is under the category of ultra-high-molecular-weight PHA [38]. The PHA isolated from the recombinant gram-negative bacteria, *E. coli* has a molecular weight of nearly 2 × 10^6^ g/mol. The polymerization and molecular weight are affected by C:N ratio [39] and are governed by the specificity of the substrate of PHA synthase [36]. The reaction temperature [36] is another vital component contributing to its polymerization and high molecular weight. Nutrient limiting media one of the essential factors contributing to PHA granule formation. Based on the subunits, sequences of amino acids, and specificity of the substrate, four classes of PHA synthase have been identified and can be correlated with the polymerization of the monomeric units to form MCL-PHA and SCL-PHA. A monomeric chain length of C3–C5 is usually noted by Class I, III, and IV that results in SCL-PHA, whereas C6–C14 chain length is often noted in Class II PHA synthase [31,40]. *R. eutropha, Pseudomonas putida*, *Allochromatium vinosum*, and *Bacillus megaterium* are representative genera of Class I, II, III, and IV [31,40]. 

*B. subtilis,* a gram-positive bacterium, has abundant peptidoglycan in the cell wall but does not have lipopolysaccharide. It allows the expression of heterologous proteins to be expressed for PHA synthesis [41]. The *Bacillus* sp. perform the dual role of polymerization and biodegradation of the SCL- PHA due to the presence of the subunits of PHA synthases (PhaA, PhaB, PhaC, and PhaR) and PHA depolymerases (PhaZ), respectively [41]. *B. cereus* is reported to synthesize unique SCL-PHA (C3–C5), (P(3HB) and P(3 HV)), when limited resources for nitrogen, potassium, sulfur, and phosphorous were provided in the nutrient media [42]. Due to the presence of both PhaC and PhaZ subunits, they are also capable of both polymerization and depolymerization of PHA [41].

Cloning experiments have been successful to unravel the mystery behind the molecular weight change. Tomizawa and his group performed an experiment considering the genes for synthesis of PHA from PHA synthase from *Bacillus* species, namely, *B. megaterium* and *B. cereus* [43]. They also considered the recombinants of these two genera and tried to express the genes in *E. coli*. They concluded that the molecular weight in P(3HB) changed due to PhaC(YB4) subunit and it was also dependent on the incubation time and the temperature. The distribution of the molecular mass in the PHA polymer, polydispersity, is also dependent on the PHA genes synthesizing polymer [38]. It was also noted that the production of the PHA granules and their molecular weight depended on that temperature when transformed from glassy material to soft material, i.e., the glass transition temperature. In recombinant *E. coli*, a feasible incubation temperature of 24–36 °C was optimum whereby the threshold temperature was close to the glass transition temperature. The thermal motion also greatly influenced the molecular weight of the polymer chain [36]. It is usually desirable to choose bacterial species yielding PHA polymer with a low polydispersity index [38] and another with high polydispersity index [44] or ultra-high molecular weight using mixed cultures so that the resultant polymer can be applicable for different industrial applications [44].

## 4. PHA as a Potential Nanocomposite 

The architectural aspects of PHA allow the production of PHA granules at the nanoscale, forming different shapes and sizes. This ability opens up wider utilization of PHA as nanocomposite. Supporting matrices for nano-sized materials and nano-biocomposites and also increasing PHA’s structural and functional properties [2]. Research suggests that one-dimensional nanostructures (nanowires, nano whiskers, and nanotubes) have been shown to be the most effective in enhancing mechanical properties due to their interdiffusion and entanglement in host polymer [45]. Another study showed that when blended with nano-bioglass and hydroxyapatite (HAP), P3HB showed tremendous improvements in sustainability to elastic stress, osteoblast compatibility, and in vivo and in vitro functioning of alkaline phosphatase [46]. Natural materials such as collagen, chitosan, hyaluronic acid, gelatin, alginate, agarose, fibrin, and cellulose are used as biomaterial scaffolds associated with PHA for spinal cord injury repair [47]. To understand the dispersion of the nanoparticles, researchers use various techniques such as X-ray diffraction, field emission electron microscopy, transmission electron microscopy, wide-angle X-ray diffraction, and simultaneous small-angle X-ray scattering [45]. Reinforcement of the PHA nanocomposites allows us to form a scaffold [48], bestowing them with multiple attributes of flexibility, mechanical strength, biodegradability, nontoxicity, and biocompatibility. These characteristic features help to enhance the applications for tissue engineering and therapeutics.

## 5. Chemical Modification for Enhancement of Functional Properties of PHA

**Figure 3:**
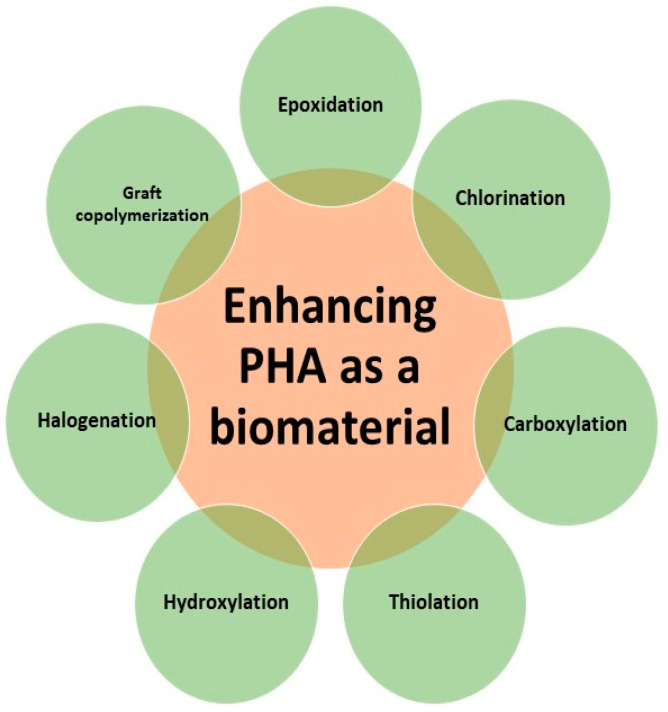
Different methods employed to increase the capability of PHA as a biomaterial. These methods modify the polymer PHA and help in crosslinking, blending, modifying, and allowing them to develop as sound nanocomposites for various applications.

Different methods are used to enhance the properties of the PHA nanocomposites (Figure 3). This is usually possible by methods such as epoxidation, chlorination, carboxylation, thiolation, and hydroxylation. Epoxidation involves the conversion of double-bonded carbon atoms into epoxides or oxiranes. This conversion can be carried out using various reagents including organic peracid, hypochlorous acid, hydrogen peroxide, etc. A recent study demonstrated the attainment of epoxy groups from the conversion of double bonds of the polymer. The double bonds caused unwanted intramolecular crosslink reactions; therefore, epoxidation was carried out. The conversion led to the obtaining of two new polymer chains, each with epoxy groups and the rate of conversion was determined to be about 80% in 1 h [49]. Chlorination is a process that involves the passage of chlorine gas through P3HB and poly(3-hydroxyoctanoate) (PHO) for modifying their properties. The molecular weight of the chlorinated samples was found to be between half and one-fourth of the original solution and the crystallinity and brittleness of PHA increased as chlorination was increased [50]. Carboxylation involves the introduction of a carboxylate functional group to the polymer. After epoxidation and chlorination, PHAs undergo carboxylation, which results in an increment in hydrophilicity. Thus, the carboxylation technique is used for controlling the degree of hydrophilicity/hydrophobicity of PHAs [50].

Thiolation is the introduction of sulfhydryl group or disulfide bonds to substrates such as PHAs for modification in their properties. This technique was used for grafting of jaffamine onto PHA that was methylated and was made to undergo thiolation using thiolactone and acetyl homocysteine (AcHC), which eventually resulted in the conversion of the amino group of jaffamine into a thiol. Using the photo grafting method, this was further grafted onto PHA. The copolymer thus obtained was thermosensitive and amphiphilic in nature. Hydroxylation employs reactions catalyzed by acids or bases in the vicinity of mono or diol compounds with low molecular weight for modifying PHA [46]. It was also reported that the kinetics of PHA oligomer production depends on the reaction conditions. The ester bonds of PHA were shown to be stable within pH 10–12 [51]. Halogenation is another method to enhance the characteristics of PHA by adding different halogens to PHA [52]. Similarly, graft copolymerization also helps in modifying PHA and providing them better mechanical characteristics [53]. All these methods help to increase biodegradability, bioactivity, flexibility, processibility, biocompatibility, and foldability.

## 6. Applications of PHA and Its Nanocomposites as a Potential Biomaterial for Medical Applications

**Figure 4:**
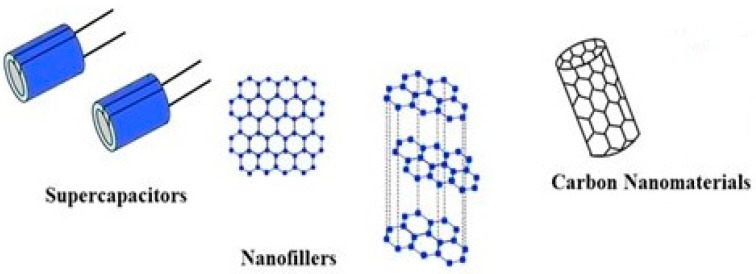
Diverse roles of PHAs as biocompatible biomaterials and as components in medical devices and varied medical applications.

PHAs and their nanocomposites offer a varied degree of polymeric properties according to their chain length and molecular weight and can be exploited for several purposes within tissue regeneration, namely, for developing sutures, wound dressings, cartilage and tendon repair, patches, bone scaffolds, etc. [15]. As a group of biocompatible and biodegradable biomaterials, PHAs have been probed for their applicability in tissue regeneration and cell lines for tissue culturing. These biopolymers lack any cytotoxicity after being implanted into an individual and this has been reconfirmed by recent research [54]. Specifically consisting of PHBHHx and P3HB4HB forms of P3HB, these polymeric nanofiber matrices also promoted the growth of HaCat human keratinocyte cell lines [55]. The significance of neuronal repairs is known because of their fineness and fragility, which makes sure that the long-distance signaling and communication between cells is undisturbed. Apart from common medical applications of PHAs, they also can be used for drug delivery systems, plasmid DNA delivery, and hormone therapy. PHA remains a crucial biopolymer as implants, bone tissue engineering, and bone scaffolds fabrication. Companies manufacturing medical devices such as biosensors, nanofillers, supercapacitors, micro- and nanochips) have also employed PHA in association with various blends such as P(3HB)/cellulose acetate butyrate [56] and PHBV/cellulose nano whiskers [57]. Nanofiber matrices of PHBHHx have been recently studied for their effectiveness in neuronal repair and results showed that these nanofibers were considered suitable for central nervous system (CNS) injury treatment and synaptogenesis of neural stem cells (NSC) [58]. Overall results of various research highlights, PHA is a prospective biomaterial for supporting tissue formation; therefore, this biomaterial holds a good chance to be considered for bone-tissue engineering [59]. Some of the promising avenues of PHA in the field of biomedical applications are discussed below in Figure 4.

### 6.1. Biosensors

**Figure 5:**
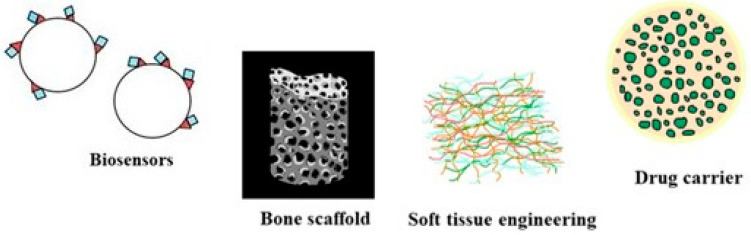
Nanocomposites of PHA as a desirable biomaterial for biosensors, bone scaffold, soft tissue engineering, and drug carrier.

PHA and its copolymers are favored for as a unique biomaterial for biomedical applications as they reduce cost and offer high good electrical conductivity [60]. PHA-graft-graphene has high electrical conductivity and an increased degradation temperature as compared to the pure form of PHA [61]. Nanomaterials based on PHA-graft-graphene can be seen in use as biomaterials (Figure 5) in nerve repair conduits, implant devices, and biosensors for their above-mentioned characteristics [62]. Moreover, PHA-graft-graphene demonstrates an increased electrical conductivity with lower critical filing content, when compared to other graphene-based blends. Lately, an electrochemical biosensor based on gold nanoparticles and an enzyme containing PHA/AuNPs/HRP/ITO was prepared with high sensitivity and selective electrochemical properties for the detection of artemisinin and spiked human serum [63]. Nanocellulose is a class of carbon nanomaterials with further two classes based on their morphology—cellulose nanofibrils (CNFs) and cellulose nanocrystals (CNCs). Carbon nanotubes (CNTs) consist of singe-wall carbon nanotubes (SWNTs) and multi-wall carbon nanotubes (MWCNTs) and because of their diverse properties, they offer a varied range of applications for PHBV matrices. MWCNTs increase the tensile strength and Young’s modulus by 88% and 172% as compared to the pure PHBV films [64]. In addition, the nanocomposites of PHBV with MWCNTs provide electrical conductivity to the nanocomposite. This resulted in an effective improvement in crystallization and nucleation over pure PHBV, demonstrating a significant increase in mechanical properties [65]. Furthermore, the composite biomaterials showed lower water absorption and water-vapor permeability. On the contrary, the use of carbon material was also reported to have a fatal toxicological effect on human and animal health. In a report by Sanpui et al. [66], an adverse report was noted with the use of SWNTs. They found pre-cancerous types of symptoms along with inflammatory responses on the use of SWNTs. They also reported an increased H1N1 infection in the lung tissue.

### 6.2. Delivery of Plasmid DNA

PHA nanocomposite serves as effective drug carriers [Figure 5] and also used for releasing proteins and nucleic acids. The delivery of plasmid DNA was carried out with the help of synthesized cationic PHA to obtain poly(hydroxyoctanoate)-co-(hydroxy-11-(bis(2-hydroxyethyl)-amino)-10-hydroxyundecanoate). The obtained polymer immobilized plasmid DNA, and it was delivered. For immobilization of proteins, inactive extracellular PHA depolymerase and MCL-PHA poly[(3-hydroxyoctanoate)-co-(3-hydroxyhexanoate)] were used [67]. Another similar study showed effective binding of acrylated monomethoxy-poly (hydroxyalkanoates) (mPHA-acrylated) with branched poly(ethyleneimine) (bPEI). mPHA-g-bPEI copolymers to siRNA, protecting it from the degradative activity of nucleases. These copolymers were engineered by mPHA-acrylated with bPEI. The blend revealed a higher transfection efficiency [68]. 

### 6.3. Packaging of Essential Hormones and Microcapsules

To ensure the delivery of pharmacologically essential agents to the desired or targeted site, engineered nanoparticle, microcapsules, and microspheres derived from PHA have been extensively probed and biocompatible systems have been developed through a few decades, at an optimal and easing rate [69]. Drugs such as anesthetics, anti-inflammatory agents, anticancer agents, antibiotics, steroids, vaccines, and hormones are intended to be delivered using various microcapsules and microspheres [70]. SCL-PHA polymers has gained substantial attention as drug carriers because of their exceptional hydrophobicity, porosity, and crystallinity, which favor the release of the encapsulated drugs without any degradation of the carrier polymer [71]. 

### 6.4. Restoration of Insulin Production and Release

PHBHHx nanoparticles (INS-PLC-NPs) were used by researchers carrying an insulin phospholipid complex. It was demonstrated by in vitro studies that there was an increased release of the insulin using the INS-PLC-NPs within 8h. In the STZ-induced diabetic rats, the hypoglycemic effect was present for over 3 days, which was highly effective as compared to the therapeutic effect of conventional administration, suggesting that the bioactivity of insulin was enhanced to many folds with the help of INS-PLC-NPs [72].

### 6.5. Microbeads for Targeted Drug Release

Several diseases such as inflammatory diseases and cancer administer PHA-derived nanoparticles and microbeads as carriers for therapeutic agents and drugs [73]. The double emulsion solvent evaporation technique has been described as a technique for developing biodegradable and biocompatible microbeads. The process includes isolation of biopolymer from *Massilia haematophila* UMTKB-2, a marine bacterium that was grown in a shake flask incubator. By removal of the endotoxins using oxidizing agents, microbeads were obtained using a peristaltic pump [74]. PHA nanoparticles serve as promising agents for targeted delivery of drugs such as Paclitaxel. They help by encapsulating the drug and transporting it to the affected tissues only and keeping the healthy cells safe from damage. Recombinant *E. coli* have also been used for loading pyrene carrying PHA nanoparticles [75].

### 6.6. Bone Marrow Scaffolds

As compared to metals and ceramics, polymers have higher processability in addition to biodegradability and biocompatibility. Therefore, they have been repeatedly probed for their use in scaffolds. When combined with nano polymers, various other nano-sized materials can be used for enhancing the interactivity of osteoblast cells with these nanostructures and boosting the cytocompatibility of the scaffolds. These polymeric nanocomposites have a greater surface area as compared to macroscopic materials, therefore, having an enhanced capacity of biomimicking bone properties [76]. There have also been several successful endeavors for designing PHA based biomaterial matrix to sustain bone tissue development on bone marrow cells. It stands out in its performance when compared to its counterpart, polylactic acid (PLA) [77].

### 6.7. Scaffolds for Bone Tissue Engineering and Processing of Biomaterials

Recent studies have highlighted PHA as an element that can be used successfully for bone scaffolds due to their properties such as biocompatibility, vascularity, and degradability [77]. It was noted that temperature had an effect on the mechanical properties of the engineered scaffolds; therefore, 20 °C and 37 °C were taken into consideration and were investigated for their effect on the scaffolds. At 20 °C, the storage capacity increased 85.6% and there was a slight increase at 37 °C with 47% and 25% increment for three and five clay loading, respectively [78]. PHA-based nanofibers have a diameter ranging between 50 nm to 500 nm, which served as perfect a mimic for collagen fibers [47,79]. Osteoconductivity and osteoinductivity can be incorporated into a bone scaffold by introducing hydroxyapatite (HAP). The scope of P3HB/HAP porous blends for bone tissue regeneration is estimated by cell viability in vitro studies as a precursor. The blends proved to be entirely sustainable and biocompatible with cell adhesion and proliferation of pre-osteoblast mammalian cells [46]. Nanofibers made up of PHBV, P3HB, PHA, and its copolymers were found to show enhanced cell proliferation and growth. These nanofibers could be conjugated by electrospraying nanoparticles of hyaluronic acid (HA). This helped to make the bone tissue porous aiding in more proliferation of the mesenchymal stem cells. Thus, regeneration of the bone extracellular matrix is enhanced. The commendably high adhesive properties of nanostructures of PHA based films comes from its hierarchical arrangement of nanoparticles throughout its polymer matrix, therefore increasing the chances of cell adhesion to many folds, due to an increased surface area and dampening of the scaffolds [80]. 

## 7. Strategies for Designing Bone Scaffolds for Bone Regeneration

PHAs as a nanomaterial serves as an excellent biomaterial for developing bone scaffolds when blended with other potential biomaterials such as hydroxyapatite, polylactic acid (PLA), polyL-lactide-co–caprolactone (PLCL), and cellulose acetate (CA). Poly 3-hydroxybutyrate-co-3-hydroxyhexanoate (PHBHHx/P(3HO-co-3HHx) and polyL-lactide-co–caprolactone (PHBHHxPCL) led to a successful adherence of osteoblast cells because of a pre-determined micro- and macro-scaffold porosity [81]. Blends of PHA with HA can give rise to nanocomposites with a strikingly good compressive strength similar to human bones [82]. PHA also shows more osteoblast regenerative properties, provides protection to stem cells against tensity, encourages the proliferation of living cells in damaged areas of the tissues, and bears enhanced adhesive properties as compared to its counterpart biomaterials [83]. Many techniques have been used for processing the PHA for biomedical use. Solvent casting particle leaching, melt molding particular leaching, fiber spinning technique (dry, wet, and melt), injection molding, phase separation, freeze-drying, gas foaming, and electrospinning are some of the common methods for making biomedical devices using PHA [81]. Some advanced strategies such as fused deposition modeling (FDM), additive manufacturing, and computer-aided wet-spinning are some more processes that are gaining good popularity for the processing of biomaterials, including PHA [81]. Selective laser sintering technique, a type of additive manufacturing technique, has been used for designing scaffolds composed of calcium phosphate (Ca-P)/poly(hydroxybutyrate-co-hydroxy valerate) (PHBV) nanocomposite, targeting a structure with high interconnectivity of pores. The nanocomposite scaffold surface was coated with gelatin and heparin followed by the amalgamation of rhBMP-2 (recombinant human bone morphogenetic protein-2). The results of this approach proved to be effective in giving rise to osteogenic cells from undifferentiated mesenchymal stem cells [84]. Some common strategies and techniques for designing bone scaffolds with hierarchical structures for bone regeneration are discussed in the forthcoming sections.

### 7.1. Solvent Casting Particle Leaching Method

**Figure 6:**
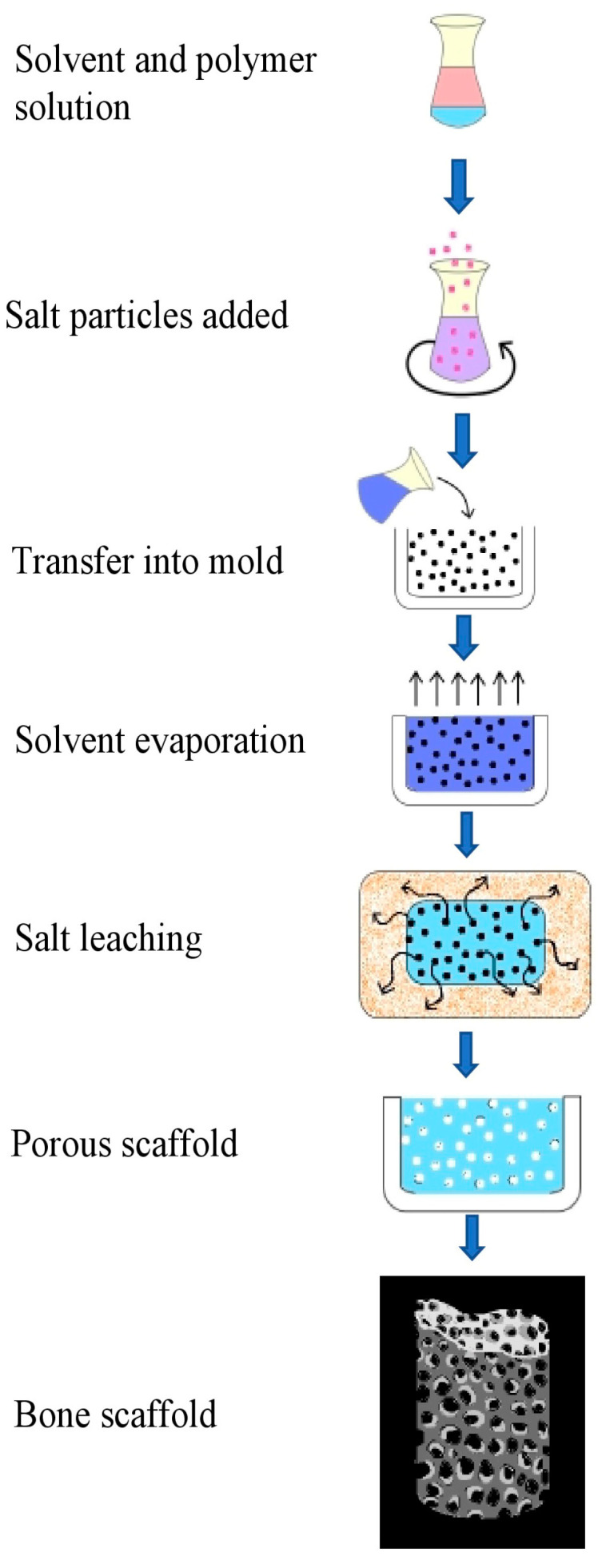
Schematic representation for designing bone scaffolds with hierarchical structures for bone regeneration using solvent casting particle leaching (SCPL) method.

The solvent casting particle leaching (SCPL) technique employs very inexpensive equipment and is known for its simplicity in terms of execution; therefore, SCPL has been a huge interest for the medical industry [85,86]. The polymers in use are introduced in a suitable solvent for obtaining a uniform polymer solution and then the insoluble salt particles are introduced to this solution serving as pore-forming agents; this mixture can further be cast into suitable molds, or salt-polymer blends could be appropriately obtained by vacuum-drying, which ensures the exclusion of organic solvent from the mixture (Figure 6). SCPL ensures that the porosity of the scaffolds is adjustable and the interconnectivity between the particles of the scaffold is adjustable by selecting different agents with varied atomic sizes [87]. When PHA-PEG scaffolds were fabricated using SCPL, with sodium chloride and polyethylene glycol (PEG) as porogens, the resultant salt-PEG leached PHA-PEG scaffold had a homogeneously distributed pore size of 378–435 μm, resulting in increased water retention and absorptivity, and is complementary to mouse calvaria-derived preosteoblastic cells with a high aggregation of mineral deposits [87]. 

### 7.2. Sol–Gel Technique

**Figure 7:**
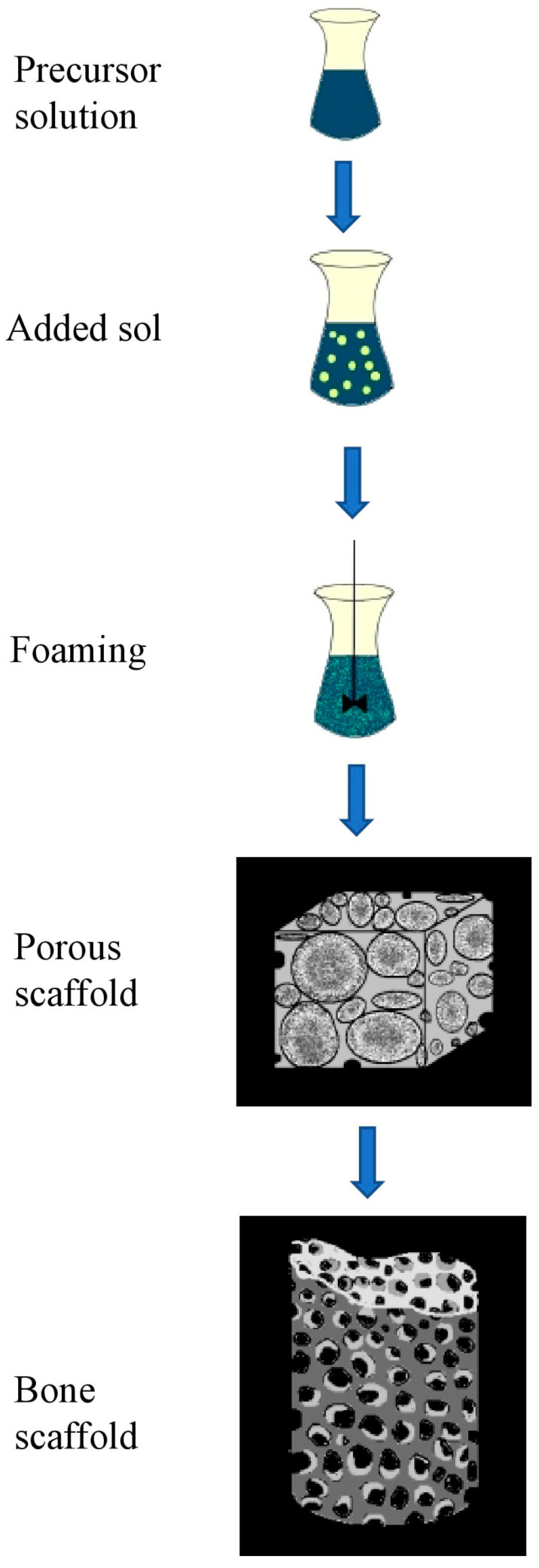
Schematic diagrammatic representation for designing bone scaffolds with hierarchical structures for bone regeneration using the sol–gel technique method.

Particles are usually mixed in the polymer matrix either by ultrasonication or by simple agitation. However, such common approaches can cause clumping or agglomeration of nanoparticles due to their high surface area and binding affinity. Therefore, sol–gel techniques are considered suitable for designing nano-glasses and implants as scaffolds. A structural composite of multiple nanomaterials can be created (Figure 7) by making mixtures of molecules in precursor solutions at a defined temperature. Hence, this technique is considered a versatile technique that ensures uniform distribution of particles throughout the polymer [88]. Bioactive nano- and micro-sized glasses are materials that are commonly obtained from sol–gel protocols, and in addition to their biodegradability, they possess characteristic features of high surface area and remarkable osteoinductivity properties. This technique is carried out by first mixing the suitable precursors, including organic or inorganic compounds (metallic), with water to give rise to sols, followed by hydrolysis reactions, the addition of surfactants, transfer of the foamed sols to molds, which finally take up the form of gels. This step is followed by thermally treating the gels to increase the overall density of the matrix [53,60,89]. The hybrid scaffolds of polyhydroxybutyrate (PHB)/PCL/58S bioactive glass (60SiO_2_-36CaO-4P_2_O_5_, mol%) are engineered by sol–gel technique. The addition of sol–gel derived bioactive glass nanoparticles could improve the scaffold stiffness significantly. The obtained sol possessed enhanced antibacterial properties in addition to biodegradability and this also upregulates osteogenic genes because of their hydrophilic properties [90].

### 7.3. Gas Foaming Method

**Figure 8:**
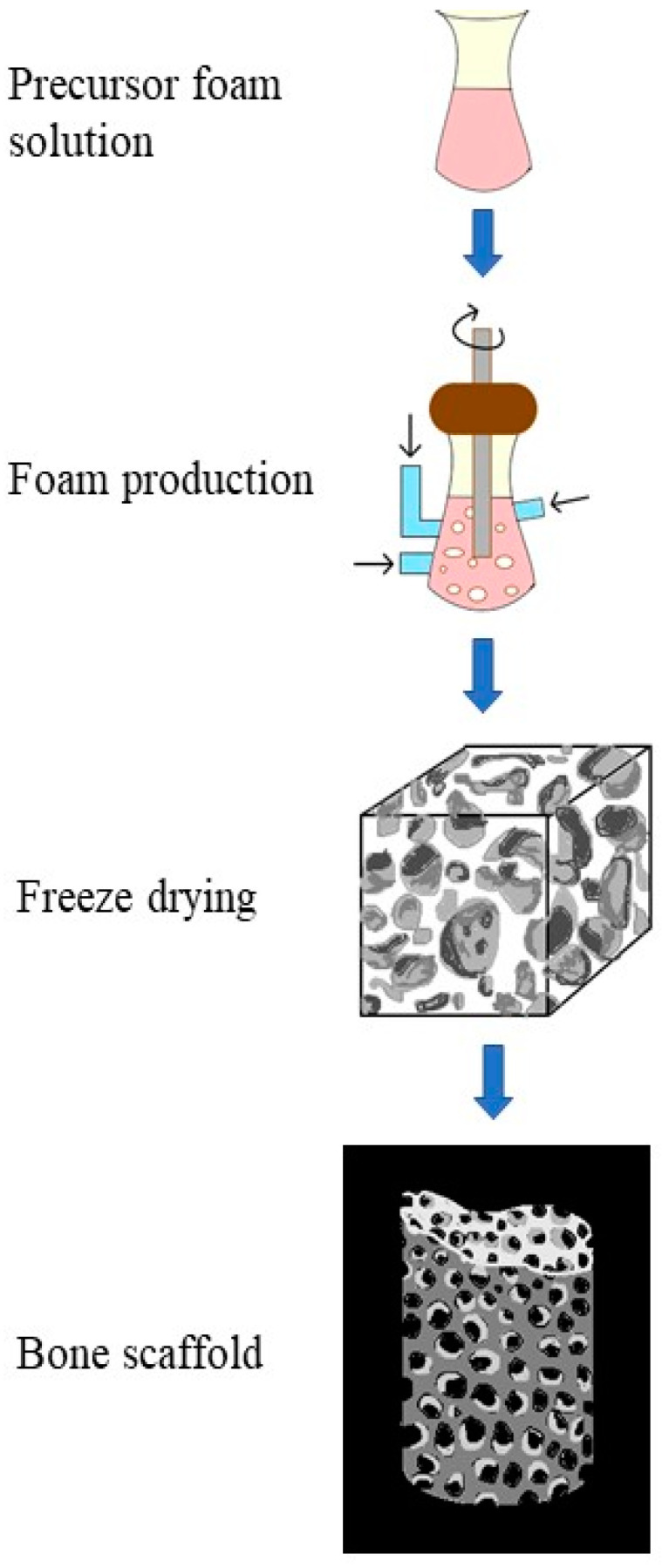
Process of fabricating a bone scaffold using PHA nanocomposite by employing gas foaming strategy.

Preparation of 3D porous structures for bone scaffolds employs gas foaming technique. Gas foaming is carried out through the bubbling of inert gases into precursor liquid solutions, and the transformation of liquid into foam is later performed [91]. In a recent study, a continuous supercritical carbon dioxide (sc-CO_2_) assisted extrusion process has been used to prepare poly(3-hydroxybutyrate-co-3-hydroxyvalerate) PHBV/organo-clays nano-biocomposite foams. The structures obtained have been characterized in terms of clay dispersion, matrix crystallization, porosity and pore size distribution, and pore density [92]. The process is initiated by mixing the substrates, the binder, and the foaming agent into an amorphous consistency, followed by the molding of this mixture into the desired shape as per the future scaffold (Figure 8). The gel is allowed to partially solidify. Gases are formed as by-products during this process, and therefore pores start forming in the material during the escape of gases. The pore size of the material developed can be regulated by adjusting the ratio of foaming agents and the constituent reagents [93]. The nano-biocomposite foams formed are more homogenous and have 50% higher porosity. This is ensured by a good clay dispersion and a highly controlled mass transfer of sc-CO_2_ [92].

### 7.4. Lyophilization

**Figure 9:**
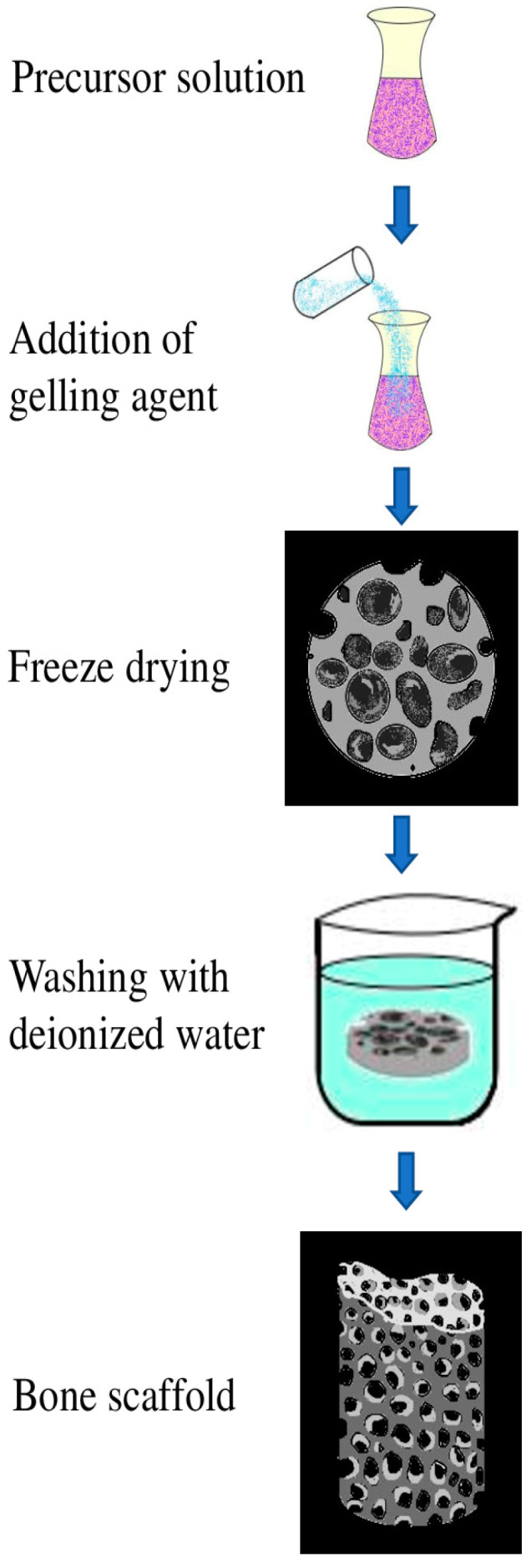
Process of fabricating a bone scaffold using PHA nanocomposite by employing lyophilization.

This method is considered to be highly versatile to obtain the desired plastic-like characteristics from PHA. This technique carries out the removal of volatile organic residues from the designed scaffolds (Figure 9). Lyophilization causes changes to the microparticles, which are irreversible and permanent, therefore making them suitable for applications in drug delivery and in bone tissue engineering [94].

### 7.5. Electrospinning

**Figure 10:**
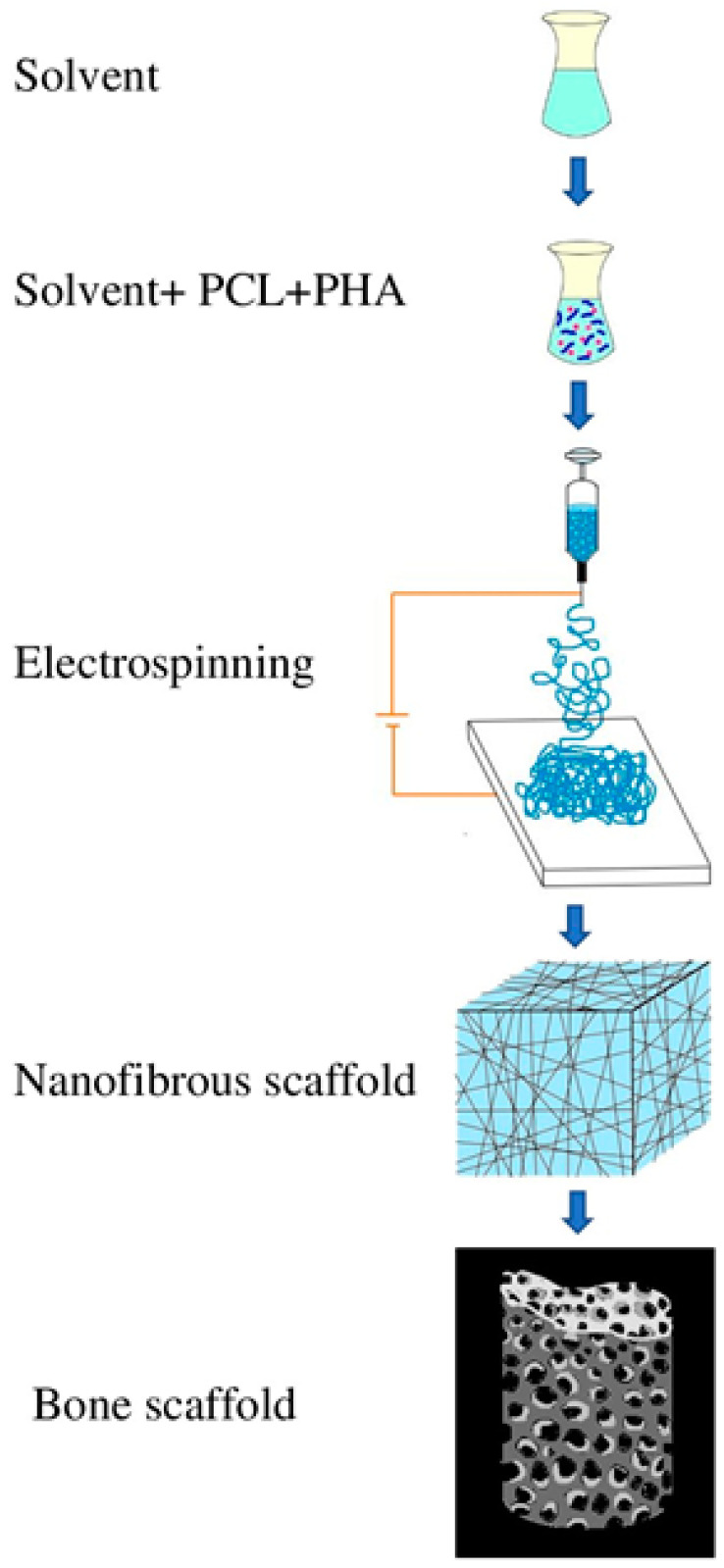
Electrospinning approach for fabricating a bone scaffold using PHA nanocomposite.

Electrospinning is a commonly used technique (Figure 10) for the fabrication of nanocomposite films [95]. Based on electrostatic attraction, electrospinning is often used for the fabrication of non-woven, long nanofibers [96]. Poly-3-hydroxybutyric acid or 4-hydroxybutyric acid are used to generate PHA nanofibers with the least cytotoxicity. They also possess more porosity between the fibers and a greater surface to volume ratio. Polyhydroxybutyrate-Chitosan (PHB-CTS) nano-scaffolds reinforced with alumina nanowires for cartilage tissue engineering were fabricated using the electrospinning method. Moreover, the addition of alumina nanowires to the PHB-CTS nanocomposite resulted in the increase of overall hydrophilicity and tensile strength of the developed cartilage scaffold [97]. Electrospinning techniques results show that MCL-PHA is not suitable for this method because they are highly elastomeric; at the same time, SCL-PHA can be electro-spun [98] but due to its brittleness, it is not suitable in medical domains where direct contact to soft tissues is involved, such as skin regeneration and drug transport [98]. SCL-PHA can be blended to form biopolymers (P3HB-HV, P3HB-CO-P4HB), terpolymer, etc. with increased mechanical properties and thus providing a wide spectrum of applications [99]. The PHA nanofibers are used for tissue engineering for nerve, liver, bone, skin, and cornea [100,101,102,103]. These PHA nanofibers can restrict the portal of entry for microbial pathogens. Nanofiber scaffolds of PHA, P3HB, and its copolymers are also used for regeneration and grafting of skin and restoring tissue damage from burns. These electrospun nanofibers of PHA can be integrated with antibacterial compounds against pathogenic organisms providing better post-remedial strategies for wound healing [104].

## 8. Future Scope and Overall Versatility of PHA as a Nanocomposite

PHA copolymers have shown more compatibility commercially than homopolymers. PHBV, a copolymer, is more elastic than P3HB, thus increasing its value in the medical utilities industry [105]. A blend of commercialized PHA and PVC has resulted in more resistance towards UV light [106]. Experimentally, copolymers such as biopolymer P(3HB/4HB), tetra polymer P(3HB/3HV/4HB), and quarter-polymer P(3HB/3HV/4HB/3HHx) have showcased an increase in the content of 4HB and a simultaneous decrease in crystalline character. Moreover, due to the addition of 3HV and 3HHx, the copolymers have developed thermal resistance, which helps in further processing that is more than P3HB [35,105]. P3HB is a universally researched homo-polymer of PHA. Its accumulation is regarded as one of the distinguishing taxonomic characteristics for bacteria. P3HB can be further co-monomerized enhancing its physical properties and decreasing crystallinity [106,107,108]. One of the limiting factors of P3HB is its brittleness and disadvantageous for becoming a substitute for conventional plastic. This limitation is overcome by the use of MCL-PHA and SCL- PHA copolymers. These polymers showcase desirable characteristics of flexibility, elasticity, less crystallalinity, and low Tm [108]. It is essential for determining its optimal survival temperature, ensuring the stability of the polymer. Experimental analysis has shown that the copolymer P(3HB-CO-3HHP) has higher stability in extreme temperatures such as 266.99 °C than homopolymers P3HB and PHHP [106]. The blending of PHA and its homopolymer and copolymer with different natural materials has been further modified and the resultant new era nanocomposites now contribute to healthcare and biomedicine industries in many ways. A commercial product as the absorbable P3HB suture “TephaFLEX “approved for use by FDA, manufactured by Tepha Inc., Cambridge, MA, created a new wave for the polymer industry [109]. Following this innovation, many industries have developed biocompatible sutures. Using the modified PH4B fiber, the product MonoMax^®^sutures, manufactured by B. Braun Surgical S.A., Barcelona, Spain, help to prevent the wound from rupturing [110]. The P4HB suture, Phantom Fiber™, (Wright Medical Group N.V., Memphis, TN, USA) is used for orthopedic surgery. Galatea surgical, Lexington, MA, USA, have manufactured scaffold using P4HB in the trade made GalaFLEX, GalaFLEX 3D and GalaFLEX 3DR scaffolds for the repair of soft tissues. Another product, BioFiber®Surgical Mesh using PHA, under the trade name of manufactured by Tornier N.V. (Tornier), Amsterdam, Netherland, is used in tendon repair in Arthroscopic surgery. Due to the thermoplasticity, rheological properties, biocompatibility, and zero cytotoxicity, many more companies are looking toward PHA and its nanocomposites as an affordable alternative to the existing plastics, carbon nanoparticles, or any other polymer. 

## 9. Conclusions 

PHA can serve as a potential biopolymer in the field of nanotechnology and nano-architectural industries. The future and scope of PHA, considering its recent introduction in bone tissue engineering, has yet a lot of ground to cover. With a great potential as a biomaterial specifically for regenerative medicine, challenges exist in the mass-scale production of these biopolymers due to their limited availability, therefore resulting in exorbitant expenses of extraction, downstream processing, and fabrication. These challenges are yet to be addressed for a more sustainable means of obtaining these eco-friendly biomaterials, which are superior candidates for the next-generation multi-fit biopolymers.

## Figures and Tables

**Table 1:** Effect of optimization on the yield of PHA (*w*/*v*%) in different strains of *Bacillus* species.

Bacterial Species	PHA Yield before Optimization (*w*/*v*)%	PHA Yield after Optimization (*w*/*v*)%	Reference
*Bacillus cereus* SS105	53.36 %	55.16 %	[18]
*B. cereus* FA11	22.66%	62.03%	[19]
*Bacillus cereus* BNPI-92	40.38%	60.67%	[20]
*Bacillus cereus* MCCB 281	58.00%	62.23 %	[21]
*B. cereus* Y23	42.7%	67.9%	[22]
*Bacillus cereus* 2156	59.30 %	68.78 %	[23]
*B. aryabhattai* PHB10	32.64%	75%	[5]
*B. drentensis* BP17	19.9%	55.5%	[6]

## Data Availability

The authors confirm that the data supporting the findings of this study are available within the article.

## Abbreviations

PHA: Polyhydroxyalkanoate; PHB/P3HB: Poly (3- hydroxybutyrate); PHBHHx/P(3HO-co-3HHx): P(3-hydroxybutyrate-co-3-hydroxyhexanoate); PHBV: Poly(3-hydroxybutyric acid-co-3-hydroxyvaleric acid)/polyhydroxybutyrate-co-valerate; MCL-PHA: Medium chain length polyhydroxyalkanoate; SCL-PHA: Short chain length polyhydroxyalkanoate; DDS: Drug delivery system; DNA: Deoxyribonucleic acid; DA: Decanoic acid; ECM: Extracellular matrix; AcHC: Acetylehomocysteine; CNS: Central nervous system; LA: Levulinic acid; PHO: Poly(3-hydroxyoctanoate); P4HB: Poly-4-hydroxybutyrate; PP: Polypropylene; FTIR: Fourier-transform infrared spectroscopy; INS: Insulin; NMR: Nuclear Magnetic Resonance; NSC: Neural stem cells; LPS: Lipopolysaccharide layer; PVC: Polyvinylchloride; SLS: Selective laser sintering STZ: Streptozotocin; EPPJ: Extracted pineapple peel juice; PLA: Polylactic acid; PLCL: PolyL-lactide-co–caprolactone; HAP: Hydroxyapatite; CNF: Cellulose nanofibrils; CNCs: Cellulose nanocrystals; CA: Cellulose acetate; CNT: Carbon nanotubes; SWCNT: Single-wall carbon nanotubes; MWCNT: Multi-wall carbon nanotubes; BG: Bioactive glasses; SCPL: Solvent casting particle leaching; HA: Hyaluronic acid; HRP: horseradish peroxidase; ITO: Indium tin oxide; NPs: Nanoparticles; PEG: Polyethylene glycol; PCL: Polycaprolactone; bPEI: poly(ethyleneimine.
